# Intimate partner violence and associated factors among HIV positive women attending antiretroviral therapy clinics in Gondar city, Northwest Ethiopia

**DOI:** 10.1186/s12905-023-02193-7

**Published:** 2023-02-01

**Authors:** Amsal Seraw Alemie, Hedija Yenus Yeshita, Ejigu Gebeye Zeleke, Birye Dessalegn Mekonnen

**Affiliations:** 1 University of Gondar Comprehensive Specialized Hospital, P.O. Box: 196, Gondar, Ethiopia; 2 Department of Reproductive Health, Institute of Public Health, College of Medicine and Health Science, University of Gondar, P.O. Box: 196, Gondar, Ethiopia; 3 Department of Epidemiology and Biostatistics, Institute of Public Health, College of Medicine and Health Science, University of Gondar, P.O. Box: 196, Gondar, Ethiopia; 4 Department of Nursing, Teda Health Science College, P.O. Box: 790, Gondar, Ethiopia

**Keywords:** Intimate partner violence, HIV positive women, Ethiopia

## Abstract

**Introduction:**

Intimate partner violence against women is a behavior within an intimate relationship that causes sexual, physical, or psychological harm to the women. It occurs among all socioeconomic, religious, and cultural groups in all settings, and affects the health of women, families, and the community at large. Determining the magnitude and determinants of intimate partner violence against HIV positive women could help to design preventive and control strategies. However, there is a dearth of information regarding the magnitude and determinants of intimate partner violence against HIV positive women in Ethiopia. Thus, this study aimed to assess the magnitude and associated factors of intimate partner violence against HIV positive women in Gondar city, Northwest Ethiopia.

**Method:**

A facility-based cross-sectional study was carried out from February to May 2021 in selected public health facilities of Gondar city among 626 HIV positive women. A systematic random sampling technique was used to select study participants. Data were analyzed using Statistical Package of Social Science (SPSS) version 20 software. Bivariable and Multivariable logistic regression models were done. Adjusted odds ratio (AOR) with a 95% confidence interval (CI) was used to identify determinants of intimate partner violence. Statistical significance was considered at a *p* value < 0.05.

**Results:**

The overall prevalence of intimate partner violence against HIV positive women within the last 12 months was 64.2% (95% CI 60.4, 68.2). Physical violence was the most common type (54.8%), followed by sexual (51.1%) and emotional (48.9%) violence. Intimate partner violence among HIV positive women was associated with women’s age 19–24 (AOR = 0.13, 95% CI 0.02, 0.79), monthly income of 500–2500 (AOR = 6.5, 95% CI 1.72, 25.0), urban residence (AOR = 0.35, 95% CI 0.13, 0.91), partner drink alcohol (AOR = 2.14, 95% CI 1.42, 4.06), and a husband with no multiple sexual partners (AOR = 0.75, 95% CI 0.34, 0.94).

**Conclusion:**

The result of this study revealed that intimate partner violence against HIV positive women was found to be high. Thus, protective measures that could increase the community’s and women’s awareness about the consequences of various forms of violence, and women empowerment are paramount. Priorities in programs of gender-based violence prevention should involve women from rural residences, older age, and males who consume alcohol.

## Introduction

Intimate partner violence (IPV) is the commonest form of violence against women and refers to any behavior within an intimate connection that causes physical, sexual, or psychological harm to those in the relationship [1]. It occurs among all socioeconomic, religious, and cultural groups in all settings [2]. Violence against women is a broad spread problem affecting women’s rights, health, and economic stability, including their families and communities [3]. It is one of the most pervasive human rights abuses, and it affects one in every three women worldwide [4]. The most common perpetrators of violence against women are male intimate partners or ex-partners [5].

According to the World Health Organization (WHO) report, overall, 35% of women worldwide have experienced intimate partner violence; out of which 30% of violence is perpetrated by partners [2]. Studies in the United States of America and Canada estimate that 68–95% of HIV-positive women experience IPV [6, 7]. In sub-Saharan Africa, the prevalence of IPV among HIV positive women ranged from 26 to 72% [7, 8]. In Ethiopia, about 34% of ever-married reproductive women have experienced some form of spousal physical, sexual, or emotional violence [9].

Intimate partner violence is associated with increased mortality, worse general health, injury and disability, chronic pain, suicide, substance abuse, reproductive disorders, and poorer pregnancy outcomes [10]. The consequences of IPV can also increase HIV/AIDS-related deaths [11]. IPV affects women’s health, exacerbates other health conditions, and decreases their capacity to make decisions on their own general health and reproductive and sexual lives [12].

Violence in all its forms can considerably increase the risk of human immunodeficiency virus (HIV) infection, predominantly in communities where traditional patriarchy, and violence against women perpetrated by an intimate partner is endorsed [13]. Evidence also indicated that violence against women may increase the risk of HIV transmission both directly and indirectly [14]. A systematic review has revealed that many people with HIV have experienced several types of IPV [7]. Coerced or forced sexual initiation has a significant contribution to a woman’s risk for HIV infection [15].

Several studies identified that IPV among HIV positive women was significantly associated with monthly income and educational status of women [16–19], religious [20], residence [9, 21, 22], number of children [23], partner alcohol and substance abuse [18, 24–26], having a partner with multiple sexual partners [18, 25, 27], partner discordant HIV status [23], suicidal thoughts [18], disclosure of HIV status [26, 28], and use of a condom or contraceptive [25, 29].

Intimate partner violence reduced women’s productivity and ability to effectively negotiate with their husbands or partners [19]. Negotiating safer sex after disclosure of HIV status to partner could induce gender-based violence. Literature indicates that HIV positive women are more likely to experience IPV compared to women who are not HIV positive [30]. Hence, integrating gender-based violence screening, control, and management activities could be a critical component of HIV prevention and care programs.

Despite the fact that determining the magnitude and determinants of IPV among HIV infected women could help to design preventive and control strategies, there is a dearth of information regarding the magnitude and determinants of IPV among HIV infected women in Ethiopia. Thus, this study aimed to assess the magnitude and associated factors of intimate partner violence among HIV positive women attending ART clinics in public health facilities in Gondar city, Northwest Ethiopia.

## Methods

### Study design and setting

A facility based cross-sectional study was conducted from February to May 2021 in selected public health facilities of Gondar city. Gondar city is located in North Gondar zone of the Amhara regional state, 750 km Northwest to Addis Ababa. According to Gondar city health department office, Gondar city has total population of 333,103 and about 78,546 women in the reproductive age group. In Gondar city, eight health facilities are providing ART services. The study was conducted in four selected public health facilities, namely Gondar Health Center, Maraki Health Center, and Azezo Health Center, and Gondar University Compressive Specialized Referral Hospital. These health facilities provide ART services for about 9894 sero-positive individuals; of which is 4236 are males and 5658 are females. Currently, a total of 5220 women of reproductive age are attending ART clinics in the selected four public health facilities [31].

### Study population

The study population was reproductive age women attending ART clinics for antiretroviral therapy in selected public health facilities in Gondar city during data collection. Those reproductive age women who were married, and HIV positive, and attending ART clinics during the data collection period were included in the study. HIV positive women who had been divorced, separated, or widowed within the previous 12 months were excluded from the study.

### Sample size calculation and sampling technique

The sample size was calculated using the single population proportion formula by taking the proportion of IPV in HIV positive women to be 46% [32], 95% confidence level (CL), 5% margin of error, and 10% possible non response, and adjusting for a 1.5 design effect, resulting 630 participants.

Initially, four public health facilities in Gondar city were selected using a simple random sampling technique (lottery method). Then, a systematic random sampling technique was applied to select the study participants by considering the number of HIV positive women visiting an ART clinic per day in each health institutions for ART service. The total sample size was proportionally allocated based on average client served in each health facility. The study population was divided by the sample size (5220/630 ≈ 8) to get the sampling interval [8]. The first woman was chosen at random as a starting point, and the data was collected every eight women until the required sample size was met.

### Study variables

#### Dependent variable

Intimate partner violence.

#### Independent variables

Socio demographic and socio-cultural factors: age, religion, educational level, partner educational level, residence, occupation, monthly income, partner occupation.

Reproductive factors: number of children, use of condom, fertility desire.

Medical characteristics: disclosure status, discordant, suicidal attempt.

Partner behavior factors: alcohol use, substance abuse and multiple sexual partners.

#### Operational definitions

Intimate partner violence is the percentage of HIV positive women who experienced physical violence, sexual violence, or emotional violence or both by their current or most recent husbands/partners in within 12 months. Having answered “yes” to at least one item in physical, or emotional or sexual violence were categorized as having faced intimate partner violence in the last 12 months [9, 33].

Physical violence by an intimate partner was defined as if women say “Yes” to one or more of aggression or physical force such as pushed or shoved, slapped or had something thrown at her that could hurt her, kicked, dragged or beaten up, hit with fist or something else that could hurt, choked or burnt on purpose, perpetrator threatened to use or actually used a gun and knife or weapon against him [9, 33, 34]. Sexual violence by an intimate partner was defined as if women say “Yes” to any of physical force to have sexual intercourse without her want to had sexual intercourse, or when she did not want to because she was afraid of what partner might do and was forced to do sexual acts that degrading or humiliating [33–35]. Emotional violence an intimate partner was defined as if women say “Yes” to any one of act such as insulted or made to feel bad about her, belittled or humiliated in front of other people, perpetrator had done things to intimidate or scare her on purpose, e.g. by yelling or smashing things, perpetrator had endangered to hurt someone she cared about [9, 33, 34].

### Data collection tool and procedure

A pretested structured interviewer-administered questionnaire was used to collect data. The questionnaire was adapted from the WHO’s multi-country study and the EDHS on women’s health and domestic violence, as well as selected other IPV related literature [9, 33, 35]. The tool was first prepared in English and then translated to local language (Amharic) and back to English to maintain its consistency. Four trained BSC nurses who are working in a health facility as data collectors and another two BSC nurses as supervisors were involved in the data collection process. Two days of intensive training was given to data collectors and supervisors by principal investigators. The instrument included socio-demographic and socio-cultural characteristics, reproductive factors, medical characteristics, and partner behavioral characteristics. The data were checked for completeness, accuracy, and consistency by the investigators on a daily basis.

### Data processing and analysis

The data were checked for completeness, consistency, and entered into Epi-info version 7.2.2. and then exported to the statistical package for social science (SPSS) version 20 software for analysis. Descriptive statistics (frequencies and percentages) were computed for all variables. The background characteristics of study participants were summarized and presented using frequency tables. Bivariable and Multivariable logistic regression analysis was done to identify determinants of intimate partner violence among HIV positive women. In bivariable logistic regression analysis, variables with a *p* value of 0.2 were fitted to multivariable analysis. Then, multivariable logistic regression was carried out to identify the independent predictors of intimate partner violence. Multicollinearity between covariates was checked using the variance inflation factor (VIF), and model fitness was checked using the Hosmer-Lemshow test. Adjusted odds ratios (AOR) with 95% confidence intervals were calculated to see the strength of association between independent variables and the outcome variable. For all statistical tests, statistical significance is accepted at *p* 0.05.

## Results

### Socio-demographic characteristics of study participants

A total of 626 women were responded completely, for a response rate of 99.4%. The mean age of the women was 32.78 (SD ± 6.27) years, with nearly half, 312 (49.8%) of them were between the ages of 25 and 34. Out of the total number of HIV positive women, 536 (85.6%) were urban. The majority, 490 (78.3%) were orthodox followers of religion. Regarding the women’s educational status, 207 (33.1%) of the HIV positive women attended primary school (Table 1).

**Table 1:** Socio demographic characteristics of HIV positive women in Gondar city, Ethiopia, 2021 (n = 626)

Characteristics	Number	Percent
Age		
19–24	55	8.8
25–34	312	49.8
35–44	234	37.4
> 45	25	4.0
Religion		
Orthodox	490	78.3
Muslim	98	15.7
Protestant	32	5.1
Catholic	6	1.0
Residence		
Urban	536	85.6
Rural	90	14.4
Women level of education		
No formal education	172	27.5
Primary education	207	33.1
Secondary education	171	27.3
More than secondary	76	12.1
Husband level of education		
No formal education	82	13.1
Primary education	183	29.2
Secondary education	203	32.4
Higher education	158	25.2
Women occupation		
House wife	295	41.7
Merchant	117	18.7
Student	18	2.9
Government employee	93	14.9
Daily laborer	97	15.5
Other	6	1.0
Husband occupation		
Farmer	70	11.2
Government employee	160	25.6
Merchant	172	27.5
Daily Laborer	151	24.1
Other	73	11.7
Monthly income		
500–2500	267	42.7
2501–5500	249	39.8
≥ 5501	110	17.6

### Reproductive and medical characteristics of study participants

Among HIV positive women, 446 (71.7%) have two to four living children. Nearly half, 285 (45.5%), of HIV positive women had no desire to have more children in the future. About, 361 (57.7%) of HIV positive women used a condom. Among the HIV positive women, 602 (96.2%) had disclosed their HIV status to their husband/partner, and 460 (76.4%) disclosed their HIV status to their partner within one week (Table 2).

**Table 2:** Reproductive and medical characteristics of HIV positive women in Gondar city, Northwest Ethiopia, 2021(n = 626)

Variables	Number	Percent
Living children		
No children	44	7.0
1	89	14.2
2–4	446	71.7
> 5	44	7.0
How many more children in the future?		
1	171	27.3
2–4	170	27.2
No more child want	285	45.5
Do use condom		
Yes	361	57.7
No	265	42.3
Partner refuse to use condom		
Yes	88	14.1
No	538	85.9
Disclose-your HIV status for your husband		
Yes	602	96.2
No	24	3.8
How long did take disclosure status		
Within one week	460	76.4
Within two week	39	6.5
Within 3–4 week	72	12
Greater than a month	31	5.1
HIV status of partner		
Positive	615	98.2
Negative	11	1.8
Have you ever thought ending your life?		
Yes	50	8.0
No	576	92
Have you ever tried to take your life?		
Yes	40	6.4
No	586	92.6

### Husband/partner behavioral characteristics

Nearly half, 303 (48.4%) of HIV positive women’ partners had drunk alcohol. Eighty-three (13.3%) of HIV positive women had partner who chew khat. Forty-seven (7.5%) of respondents’ partner were smoking for cigarette. Among the study participants, 125 (20%) of women’s husband had multiple sexual partners.

### Prevalence of intimate partner violence

**Fig. 1:**
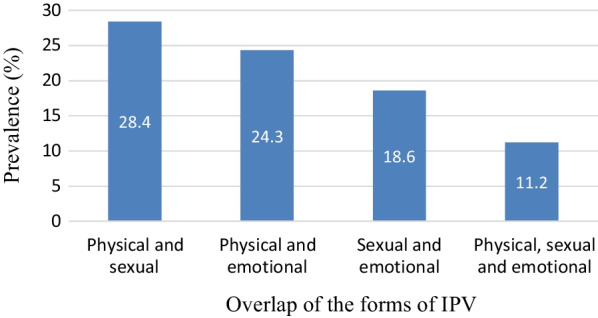
Overlap of the three most common forms of IPV among HIV positive women, 2021

The overall prevalence of intimate partner violence within the last 12 months against HIV positive women was 64.2% (95% CI 60.4, 68.2). Physical violence was the most common form, with a prevalence of 54.8% (95% CI 50.8, 58.8), followed by sexual violence 51.1% (95% CI 47.3, 55.5) and emotional violence 48.9% (95% CI 44.9, 52.7). The most frequently overlapping occurrence of IPV was physical and sexual (28.4%), followed by physical and emotional (24.3%) and sexual and emotional violence (18.6%). The joint occurrence of all forms of IPV was 11.2% (Fig. 1).

Of the acts of physical violence, the act of slapping or throwing something at an HIV positive woman was cited as the commonest form of violence, with a prevalence of 46.6%. The commonest form of sexual violence was physically forcing HIV positive women to have sexual intercourse (44.2%), whereas insulting or feel bad to HIV positive women was reported as the most frequent act of emotional violence, with a prevalence of 42% (Table 3).

**Table 3:** Percentage distribution of the type of IPV among HIV positive women attending ART clinic in Gondar city, northwest Ethiopia, 2021

Types of violence	Number	Percent
Physical violence		
Slapped or thrown something	292	46.6
Pushed or shoved or pulled your hair	220	35.1
Hit you with his fist	84	13.4
Kicked or dragged or beaten you up	92	14.7
Choked or burnt on purpose	22	3.5
Threated with or used weapon	46	7.3
Sexual violence		
Physically forced sexual intercourse	277	44.2
Had sexual intercourse because of afraid	240	38.3
Forced to something sexually degrading or humiliating	176	28.1
Emotional violence		
Insulted you or made you feel bad	263	42
Threatened to hurt you or someone you care about	83	13.2
Belittled or humiliated you in front of other	75	12
Scare or intimidate you	98	15.7

### Factors associated with intimate partner violence among HIV positive women

After the variables were screened using a bi-variable analysis with a *p* value ≤ 0.2 as a cutoff, sixteen variables, including residence, women’s level of education, husband’s level of education, monthly income, partner’s drinking alcohol, partner’s chewing of khat, partner cigarette smoking, refusal to use a condom, husband’s HIV status, women’s suicidal attempts, age, frequency of alcohol drinking, number of living children, how long disclosure status, husband with multiple sexual partner, and future plan to have children, were entered into a multi-variables logistic regression model. Age of women, residence, partner drink alcohol, monthly income, and husband with multiple sexual partners (extra marital sex) were variables significantly associated with intimate partner violence among HIV-positive women in a multivariable logistic analysis.

When compared to HIV positive women aged 45–49, HIV positive women aged 19–24 years were 87% (AOR = 0.13, 95% CI 0.02, 0.79) less likely to experience intimate partner violence. HIV-positive women living in cities were 65% less likely to have IPV than HIV-positive women living in rural areas (AOR = 0.35, 95% CI 0.13, 0.91). Intimate partner violence was 6.5 times more likely among HIV positive women with a monthly income of 500–2500 (AOR = 6.5, 95% CI 1.72, 25.0) than among women with a monthly income of more than 5501.HIV positive women whose intimate partners consumed alcohol were 2.14 times (AOR = 2.14, 95% CI 1.42, 4.06) more likely to experience intimate partner violence than HIV positive women whose partners didn’t consume alcohol. HIV positive women whose partners had non-multiple sexual partners were 25% less (AOR = 0.75, 95% CI 0.34, 0.94) likely to experience intimate partner violence than those whose partners had multiple sexual partners (Table 4).

**Table 4:** Factors associated with intimate partner violence among HIV positive women attending ART clinic in Gondar city, northwest Ethiopia, 2021

Variables	Intimate partner violence		COR (95% CI)	AOR (95% CI)
	Yes	No		
Age				
19–24	35	20	1.47(0.52, 4.12)	0.13(0.02, 0.79)*
25–34	196	116	1.52(0.62,3.75)	0.32(0.72, 1.40)
35–44	153	81	1.36(0.55,3.39)	0.57(0.14, 2.24)
45–49	18	7	1	1
Residence				
Urban	330	206	0.4 (0.31, 0.8)	0.35(0.13, 0.91)*
Rural	72	18	1	1
Women level of education				
No formal education	117	55	1.23(0.68, 2.23)	0.72(0.17, 2.99)
Primary education	128	79	1.62(0.91, 2.88)	0.18(0.51, 6.53)
Secondary education	102	69	1.78(0.98, 3.12)	1.95(0.58, 6.61)
More than secondary	55	21	1	1
Husband level of education				
No formal education	59	23	0.87(0.48, 1.56)	0.54(0.14, 2.15)
Primary education	109	74	1.51(0.96, 2.36)	0.82(0.27, 2.49)
Secondary education	125	78	1.39(0.89, 2.15)	0.58(0.22, 1.53)
More than secondary	109	49	1	1
Monthly income				
500–2500	165	102	2.21(1.32, 3.71)	6.56(1.72, 25.0)*
2501–5500	151	98	2.33(1.38, 3.91)	6.99(2.01, 24.2)
> 5501	86	24	1	1
Partner alcohol drink				
Yes	208	95	1.46(0.49, 1.95)	2.14(1.42, 4.06)*
No	194	129	1	1
Frequency of partner drink alcohol				
Sometimes	44	31	1	1
Usually	58	31	1.32(0.70, 2.48)	1.09(0.51, 2.33)
Always	107	33	0.58(0.32, 1.04)	0.58(0.29, 1.15)
Partner chew chat				
Yes	71	12	3.8(2.01, 7.16)	0.43(0.10, 1.83)
No	331	212	1	1
Partner cigarette smoking				
Yes	43	4	6.6(2.33, 18.6)	5.77(2.09, 24.6)
No	359	220	1	1
Partner with multiple sexual partner				
Yes	32	93	1	1
No	192	309	0.55(0.36, 0.86)	0.75(0.34, 0.94)*
Husband refuse to use condom				
Yes	24	64	0.63(0.38, 1.05)	0.53(0.23, 1.23)
No	200	338	1	1
Husband HIV status				
Yes	396	219	1.51(0.46, 4.99)	2.33(0.35, 15.7)
No	6	5	1	1
Suicidal attempts				
Yes	33	7	2.8(1.21, 6.38)	1.09(0.32, 3.47)
No	369	217	1	1
Disclose HIV status for husband				
Yes	388	214	1.3(0.57, 2.96)	2.57(0.60, 11.01)
No	14	10	1	1
Number of living children				
No children	19	25	3.51(1.44, 8.56)	2.42(0.97, 16.7)
1	54	35	1.73(0.79, 3.80)	1.51(0.36, 6.24)
2–4	297	152	1.36(0.68, 2.72)	0.75(0.24, 2.33)
> 5	32	12	1	1
How many more children in future?				
1	108	63	1.13(0.76, 1.68)	0.85(0.43, 1.69)
2–4	106	64	1.17(0.79, 1.74)	0.99(0.42, 2.37)
No more child want	188	97	1	1

*COR* Crude odds ratio, *AOR* Adjusted odds ratio, *CI* Confidence interval, *1* reference category, *p < 0.05

## Discussion

Intimate partner violence against HIV positive women is a common public health problem globally, and its prevalence varies from setting to setting [36]. The present study was carried out to determine the magnitude and determinants of intimate partner violence among HIV positive women who were attending ART clinic in public health institutions in Gondar city. Accordingly, more than three-fifths of HIV positive women experienced different forms of IPV. This finding implies the necessity for empowering women in their struggle toward the elimination of IPV, creating awareness for men, and prompting punishment of the perpetrators to end all the forms of IPV.

In this study, the overall magnitude of intimate partner violence against HIV positive women within the last 12 months was 64.2% (95% CI 60.4, 68.2). This result is comparable to the findings from Wolayita and Jimma in Ethiopia [25, 37] and Togo [18], which reported the prevalence of IPV as 61.3%, 64.7%, and 63%, respectively. This finding was significantly higher than in previous studies conducted in Adama town, Ethiopia (32.3%), Baltimore, America (26.5%), Nairobi and Mombasa, Kenya (20% and 14.6%), and India 35.4% [20, 27, 29, 38, 39]. This finding was lower than studies done in different parts of the Awi zone, Ethiopia (78%), Nekemet, Ethiopia (72.5%), the WHO multi country study (71%) [37, 40, 41], and a study done in Kenya 76% [42]. This variation may be due to variation in socio-economic status, reproductive health service coverage, and other gender related health services. Another variation could be due to the measurement tool for the traumatic life events questionnaire in American study (TLEQ) [17].

The prevalence of emotional intimate partner violence, 48.9%, reported in this study was found to be lower than the finding that was reported in South Africa 55.1% [24]. In addition, the reported sexual violence was lower than the study done in Togo 69.7% [18]. The difference may be explained by their different cultures, and the probable cause of the discrepancy may be owing to the difference in the small sample size in Togo and the fact that they used a different measurement tool in South Africa (an audio computer assisted self-interview) [18].

In the current study, factors associated with IPV among HIV positive women were identified. Accordingly, study participants who were from urban residences were less likely to experience IPV than those of who were from rural residences. Similar findings were documented from previous studies conducted in Ethiopia, Gondar, and Debremarkose [9, 21, 22]. The reason could be that women who are from rural areas may be influenced by traditional influences and gender norms that support wife beating and accept this catchment area, because they might not have access to information that deals with violence reduction mechanisms, and women’s rights of equality with their intimate partner.

The results of this study revealed that women aged 19–24 years were less likely to experience intimate partner violence than study participants whose age was 45–49 years. This is in line with a study conducted in Debremarkose, Ethiopia [9, 21]. The reason could be explained by the fact that family size increases as the age of women increases, which may result in an economic crisis that leads to spousal disagreement [9].

This study also found that HIV positive women with a monthly income of 500 to2500 were about six times more likely to contract IPV than women with a monthly income of more than 5501. This finding was consistent with previous studies conducted in Ethiopia [9, 29]. The possible reason could be that women who are economically dependent and who believe in the myth of male superiority are less able to leave abusive partners, resulting in prolonged exposure to IPV. This indicated that women in low income households had a higher rate of intimate partner violence [9].

The findings of this study showed that HIV positive women whose partners drink alcohol were about two times more likely to experience IPV by their partners, which is consistent with previous studies in Ethiopia [25, 43], and Rwanda [29]. This could be due to the fact that alcohol consumption can alter mental judgment, and cause aggression, which further increases the likelihood of violence. Furthermore, some people may intentionally use alcohol to hide behind it in order to engage in anti-social behaviors such as violence against their partners, which increases the possibility of violence [44].

The findings of this study identified that HIV positive women whose partners were not engaged in multiple sexual relationships were less likely to experience IPV. This is supported by studies done in Ethiopia [29], Togo [18], and Nigeria [26]. This could be attributed by the fact that some communities’ privileges men having more rights and power over women in sexual relationships. In the other hand, women who believe that their husbands have an extra-sexual partner may react violently toward a man out of jealousy, and men may use violence in response to their partner’s accusation of infidelity, which leads to women violence due to the existence of traditional gender norms that support wife beating and accept this catchment area [18].

This study provides vibrant evidence to inform policy-makers and other relevant stakeholders about how to prevent and control IPV among HIV positive women. Some factors have been identified as being associated with increased IPV experiences among HIV positive women. As a result, prioritizing the factors and initiating IPV prevention should begin sooner rather than later. A variety of forms of IPV were identified that are commonly experienced among HIV positive women. Thus, education, training, and information provision programs are essential to assist and empower HIV positive women.

### Strength and limitation of the study

The strength of this study is, the use of validated instruments from a multi-country WHO questionnaire on intimate partner violence against women. However, the study has some limitations that should be considered when interpreting the results. Since the topic is sensitive, some respondents may not be willing to disclose their violence, which may lead to underreported (social desirability bias). To reduce this limitation, a separate room was prepared for interviewing each woman. Every possible effort was made, and well-trained female data collectors were used so as to increase disclosure of information. In addition, attention was given in the wording of questions in each section of questionnaires by forewarning about the sensitive nature of the questions and by ensuring that the information should be kept secret.

## Conclusion

The result of this study identified that intimate partner violence against HIV positive women was high. Physical violence was the most common type, followed by sexual and emotional violence. Intimate partner violence was more likely among HIV-positive women with low monthly incomes and alcoholic partners. HIV positive women from rural areas, who were younger, and whose partners did not have multiple sexual partners were less likely to experience intimate partner violence. Thus, protective measures that could increase the community’s and women’s awareness about the consequences of various forms of violence and women’s empowerment should be taken with the collaborative effort of policymakers, programmers, healthcare providers, and other stakeholders. Priorities in gender-based violence prevention programs include women from rural residence, older people, and men who drink alcohol, are paramount. In addition, further study using a qualitative approach is required to clearly understand all types of abuse.

## Data Availability

The datasets used and analyzed during the current study is included in this article.

## Abbreviations

AHR: Adjusted hazard ratio
ART: Antiretroviral therapy
AIDS: Acquired Immune Deficiency Virus
CI: Confidence interval
EDHS: Ethiopian Demography Health and Survey
HIV: Human Immune Virus
IPV: Intimate partner violence
SPSS: Statistical Package for Social Science
STI: Sexual transmitted infection
WHO: World Health Organization
